# Adherence to insulin self administration and associated factors among diabetes mellitus patients at Tikur Anbessa specialized hospital

**DOI:** 10.1186/s40200-017-0309-3

**Published:** 2017-07-01

**Authors:** Yusuf Gerada, Zuriyash Mengistu, Asrat Demessie, Atsede Fantahun, Kahsu Gebrekirstos

**Affiliations:** 1 Addis Ababa University, Tikur Anbessa Specialized Hospital, Addis Ababa, Ethiopia; 2 Department of Nursing, College of Health Sciences, Addis Ababa University, Addis Ababa, Ethiopia; 3 Department of Nursing, College of Health Sciences, Mekelle University, Mekelle, Ethiopia

**Keywords:** Adherence, Insulin self administration, Diabetes mellitus

## Abstract

**Background:**

The goals of diabetes treatment are to keep blood glucose levels as near normal as possible while avoiding complications. Despite the benefits of insulin therapy, many people with diabetes don’t adhere to treatment. Some avoid insulin therapy or refuse to start it. Several studies investigating adherence to chronic disease treatment have evidenced that patients often discontinue their medications or even do not take them at all because they consider them ineffective or experience untoward side effects. To assess adherence to insulin self administration and associated factors among adult patients with diabetes mellitus at endocrinology unit of Tikur Anbessa Specialized Hospital Addis Ababa Ethiopia.

**Methods:**

A cross-sectional study was conducted from December to June 2015, on a total of 378 diabetic patients on insulin self administration using convenience sampling method. The data was collected using structured questionnaires after ethical approval and informed signed consent have been taken. The data entry and analysis was conducted using Epi info version 3.5.4 and SPSS version 21.

**Results:**

One hundred twenty five (33.1%) of the respondents were found to be non-adherent to insulin self injection. Multivariate analysis identified who stopped taking insulin when they feel better, who have Heart disease and those not taking insulin when they were out of home for long time as independent factors for non adherence of insulin self administration.

**Conclusion:**

The factors associated with non adherence to insulin self administrations were; forgetting time of injection, deliberately, feeling better and feeling worse.

## Background

Diabetes mellitus (DM) is defined as a group of metabolic diseases characterized by hyperglycemia (increased blood glucose level) resulting from defects in insulin secretion, insulin action, or both [1]. Globally Diabetes becomes a significantly growing health problem and it is also an important problem in Africa. It is chronic disease that needs long term medical care and follow-up to prevent complications associated with the diseases [2]. Chronic hyperglycemia is associated with long-term damage and failure of different organs of the body, especially the eyes, kidney, nerves, heart and blood vessels [3]. The patients with DM are also at a high risk of acute complications like hypoglycemia [4]. Adherence to treatment significantly influences the prevention and control of acute and long-term complications of DM [5]. Low cost strategies that can reduce the impact of diabetes and associated complications are: lifestyle modifications, physical activity and effective use of medication [6].

The goal of diabetes treatment is mainly to control blood glucose levels as near normal as possible with minimal complications [7]. In contrary to the benefits of insulin therapy, significant number of patients with diabetes show low adheres to treatment and some patients avoid insulin therapy or not willing to start it [8]. Several studies about chronic disease treatment have showed that patients discontinue their medications or even do not take them at all because they perceive that they are ineffective with untoward side effects [9–11]. Recent reports from World health organization (WHO) shows alarming magnitude of non-adherence and its long term complications, therefore it is very important improving adherence to existing treatments than developing new treatment modalities [1]. In developing countries effective insulin administration to manage hyper glycaemia remains challenging [12]. In most developing countries like Ethiopia, insulin is commonly available as single or multiple subcutaneous doses every day and the commonly used insulin injection devices are insulin syringes and pens. Insulin therapy remains unacceptable amongst patients with DM because of different reasons like needle phobia, expenses and inconvenience of the daily injections [12].

Even though different factors can affect treatment adherence, it is remains difficult to identify the main factors associated with treatment adherence. First, access of patients to drugs needs due consideration to rule out possibility of inaccessibility of the drugs [13]. Factors that associated with patient adherence to drug therapy can be grouped as follows: patient-related; patient-provider relationship, therapeutic regimen, and the disease itself [13]. Primary prevention of risk factors and secondary prevention of adverse health outcomes are some of the interventions that can help to improve adherence which in turn positive outcomes on the health condition of patients [14].

To improve patient adherence, it is important to understand the extent of patient adherence and why non-adherence to insulin self administration occurs. Even though there are studies conducted in different parts of the world, there is no enough studies conducted in the study area. Therefore, the objective of this study was to assess the pattern of adherence to insulin self administration and associated factors among adult patients with diabetes mellitus at endocrinology unit of Tikur Anbessa Specialized Hospital Addis Ababa Ethiopia.

## Methods

The study was conducted from December to June, 2014 at Endocrinology unit of Tikur Anbessa Specialized Hospital, Addis Ababa City which is the main tertiary referral teaching and research Hospital in Ethiopia. Hospital based cross-sectional study design was used to conduct this study. The inclusion criteria was: having diabetes either type 1 or type 2, non-pregnant, aged at least 18 years attending the diabetic clinic during the study period, taking insulin by themselves and giving written informed consent to participate in the study. Convenient sampling technique was used to Select 378 patients with DM who meet the mentioned inclusion criteria. The sample size was calculated using single population proportion formula (n = (Zα/2) ^2^ p(1-p)/d ^2^). Data was collected using interviewer administered structured questionnaire which was developed from different literatures. The questionnaire was pretested in 10% of the sample size in different institution which was not included in the study to assess the validity and reliability. Non-adherence was assessed using patients self reports of how they had been taking their; Patients were asked to recall if they missed any doses of medication on a day by day basis over a period of one month. The number of injection missed was calculated based on the prescribed dose. Patients who reported taking less than 80% of their prescribed insulin injection was considered as non adherent to treatment. Clarity and completeness of the filled questionnaire was checked daily during the data collection period. Data was entered to Epi-info version 3.5.4 and exported to SPSS version 21.0, then cleared and analyzed. Descriptive statistics was computed to show frequency, percentage distribution, mean, median, range and standard deviation. Appropriate statistical techniques for data analysis was applied to determine association (OR and/or chi-square). Statistical significance was evaluated at 95% level of significance and the result was presented in the form of tables and pie chart.

## Results

### Socio demographics characteristics

A total of 378 respondents participated in this study with response rate of 100%. Majority of the respondents were found to be at the age category of between 31and 55 years which accounts 195(51.6%), the minimum and the maximum age of respondents was 19 and 78 years old respectively. One hundred ninety three (51.1%) participants were males and 343((90.7%) of the respondents were urban residents. Two hundred sixty one (69.0%) of participants were married and Orthodox in religion. Regarding educational status, 136(36.0%) were in Secondary education [9–12], as well as 126(33.3%) patients had monthly income of 500–1000 Birr (Table 1).

**Table 1:** Socio-demographic distribution of insulin self administration diabetes patients in Endocrinology unit of TASH 2014 (*n* = 378)

Variables		Frequency	Percent
Age	≤30	109	28.8
	31–55	195	51.6
	56–80	74	19.6
Sex	Male	193	51.1
	Female	185	48.9
Residence	Urban	343	90.7
	Rural	35	9.3
Ethnicity	Amhara	147	38.9
	Oromo	97	25.7
	Guraghe	59	15.6
	Tigre	40	10.6
	Others	35	9.3
Marital status	Married	261	69.0
	Single	75	19.8
	Widowed	20	5.3
	Others (divorced, cohabitation)	22	5.8
Religion	Orthodox	248	65.6
	Muslim	82	21.7
	Protestant	34	9.0
	Catholic	8	2.1
	Others (pagan, Johba and Wakefata)	6	1.6
Level of education	No formal education	32	8.5
	Primary education [1–8]	108	28.6
	Secondary education [9–12]	136	36.0
	Diploma and above	102	27.0
Occupation	Business/Self employed	124	32.8
	Government employee	86	22.8
	Housewife	68	18
	NGO/Private sector employee	29	7.7
	Student	22	5.8
	Farmer	19	5.0
	Others^a^	30	7.9
Monthly income	<500	110	29.1
	500–1000	135	35.7
	>1000	126	33.3
	Unknown	7	1.9

^a^Daily workers, retired

### Diabetes history

About half 186 (49.3%) of participants’ duration of living with diabetic was greater than 10 years. One hundred seventy three 173 (45.8%) of patients started insulin self administration therapy since 5–10 years. About 366 (96.8%) clients used insulin syringe for self injection. Almost all (97.4%) of participants’ injection schedule of insulin was twice a day. One hundred ninety five (51.6%) participants have been using one needle for 2–6 days and only 4 (1.1%) of participants have been using single needle once as recommended. More than half 261 (69.0%) of patients visited to health care provider once every 6 months, only 17 (4.5%) visits health care provider once a month. Two hundred fifty seven (68.0%) of patients provided insulin freely from the Tkur Anbesa Specialized Hospital endocrinology unit. All most all of 371 (98.1%) had regular follow up of DM clinic (Table 2).

**Table 2:** Distribution of diabetes patient by duration with diabetic and insulin self administration in TASH, 2014

Variable		Frequency	Percent (%)
Duration with Diabetes Mellitus	<10 (Below median)	136	36
	10 (Median duration)	56	14.8
	>10 (Above median)	186	49.3
Duration of insulin therapy (self administration)	1–4 years	107	28.3
	5–10 years	173	45.8
	≥11 years	98	25.9
Type of insulin device	Insulin syringe	366	96.8
	Insulin pen	8	2.1
	Needle with separate syringe	4	1.1
Dosing schedule of insulin	Once a day	7	1.9
	twice a day	368	97.4
	more than 2 times per day	3	0.8
Duration of needle use	Once	4	1.1
	One day	6	1.6
	2–6 days	195	51.6
	7 or more days	173	45.8
Regular follow up	Yes	371	98.1
	No	7	1.9
Duration of visit to health care provider	Once in a month	17	4.5
	Once in 3 month	100	26.5
	Once in 6 month	261	69.0
Source of insulin	Free	257	68.0
	Purchase	121	32.0
Cost of insulin	Costly	374	98.9
	Cheap	4	1.1
Chronic condition(s) patient have^a^	Hypertension	164	43.4
	Heart diseases	60	15.9
	High cholesterol	58	15.3
	Lung disease	8	2.1
	Others diseases	31	8.2

^a^More than one answer was given

### Practice of insulin self administration

**Fig. 1:**
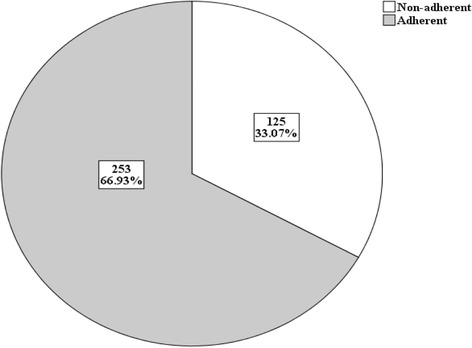
Distribution of diabetes patients by adherence and non adherence to insulin self administration in TASH in endocrinology 2014. (*n* = 378)

Almost all (99.2%) respondents diagnosed as having DM for the first time by physicians. One third (33.07%) of the respondents are found to be non-adherent to insulin injection (Fig. 1). only one in every 10 participants took his/her insulin injections at the same time daily as recommended while 347 (91.8%) did not take insulin on similar time daily. Only 31 (8.2%) participants did have ever missed doses of their insulin for some reasons; among these difficulty to afford the cost of insulin 4 (1.1%) and due to nature of their work/schedule of work 15 (4.0%). Twenty six (6.9%) of participants stopped taking their medication when they were feeling better. Two hundred sixty three (69.6%) were using abdomen as site of injection and 276(73.0%) used upper arm. About 65.1% Respondents store/keep their insulin in refrigerator. About three fourth (75.7%) of participant took their insulin with themselves when they were out of home for long time.

#### Factors associated with insulin self administration

Multivariate regression analysis identified the factors associated with adherence to insulin self administration: those who stopped taking insulin when they feel better, those who have Heart disease and those taking insulin when they were out of home for long time (Tables 3 and 4).

**Table 3:** The crude odd ratio (COR) that predict adherence to insulin self administration of diabetes patients in Endocrinology unit of TASH 2014 (*n* = 378)

	Sig.	COR	95% C.I. for COR	
			Lower	Upper
Farmer	0.030	0.188	0.041	0.850
Business/Self employed	0.013	0.202	0.057	0.710
Student	0.008	0.136	0.031	0.592
Heart diseases	0.003	2.347	1.341	4.110
Who stopped taking insulin when they feel better	0.023	0.395	0.177	0.881
Insulin is expensive	0.031	11.40	1.256	103.510
Adverse reaction at insulin injection site	0.006	0.536	0.344	0.836
Taking insulin when they were out of home for long time	0.015	1.819	1.121	2.951
Who encountered local irritation	0.031	0.622	0.404	0.959
Who developed Lipohypertrophy	0.029	0.595	0.374	0.947

**Table 4:** The adjusted odd ratio (AOR) that predict adherence to insulin self administration of diabetes patients in Endocrinology unit of TASH 2014 (*n* = 378)

Ever missed taking insulin injection	Sig.	AOR	95% CI for AOR	
			Lower	Upper
Intercept	0.001			
Occupation	0.246	1.068	.956	1.194
Who have Heart disease	0.001*	2.647	1.470	4.766
Who stopped taking insulin when they feel better	0.005*	3.309	1.423	7.698
Adverse reaction at injection site	0.050	1.663	1.001	2.765
Taking insulin when they were out of home for long time	0.031*	0.565	.336	.948
Who encountered local irritation	0.789	0.927	.533	1.614
Who developed Lipohypertrophy	0.104	1.577	.911	2.729

*Level of significance ≤ 0.05

## Discussion

This study tried to identify the factors associated with non adherence to insulin self administration. About 26 (6.9%) of respondents stopped taking their medication during better feeling and 12 (3.2%) during worse feeling. This finding is similar with study done in French [15]. In this study about 96.8% participants use insulin syringe and the rest use insulin pen and needle with separate syringe. This was higher as compared to a study conducted in India which showed 68.1% patients used Insulin syringes [16]. This difference might be due to time gap and the availability of insulin syringes.

In this study among about 64.3% respondents were developed adverse effects like lipohypertrophy at injection site. Whereas in a study conducted in Spain found that lipohypertrophy at injection sites complained by 29% patients [17]. This might be due lack of awareness and practice about injection site rotation. In this study 69.6% of respondents were commonly used abdomen and 86% upper thigh as injection site. In study conducted in Spanish Similarly abdomen and thigh of the participants had used as common injection sites [17].

In this study The factors associated with non adherence to insulin self administration were feeling better, pt developed heart disease and pt went out of home for long time. Where us in a study done in Tehran, Iran, Factors that showed a significant association with insulin compliance were: being a time consuming process; embarrassment; feeling worse after injections; forgetfulness; sick days; experience of hypoglycemia; medication cost; weight gain; insulin shortage; and difficulties in preparing injection [18]. This difference might be because of variations in patient’s awareness, analysis methods and study design.

In a study done in south India on the other hand, factors such as illiteracy, economic problems to buy medications, lack of information on prescribed medications, lack of awareness on the importance of regular medications, not visiting physician regularly and not following advice on diet are the major ones affecting non-adherence [19]. This variation might be due to difference in health care delivery system of the two countries.

In a study conducted in Adama Referral hospital, Ethiopia the factors associated with non adherence were reported as patient related such as (forgetfulness, intentional omission of dose) and drug related (cost, side effects, and multiple drug therapy especially in those with comorbidity) [20]. This difference might be due difference in Sociodemographic characteristics of respondents.

## Limitations

- The study was mainly quantitative cross sectional, it cannot assess other possible factors of non adherence than those mentioned in literatures
- The study area was only Addis Ababa, this make difficult to generalize at community level

## Conclusion

Based on the findings of this study, the following points concluded:

- One third of the respondents were found to be non-adherent to insulin injection. This is significant number which needs intervention to minimize the immediate and late complications associated with non adherence to insulin. So, ministry of health and significant others are recommended to act to minimize the non adherence level
- The factors associated with non adherence of insulin self administration were feeling better, pt developed heart disease and pt went out of home for long time as. These factors can be minimized through continuous monitoring and patient education by health professionals working in Endocrinology unit.

## Abbreviations

AOR: Adjust odds ratio
COR: Crude odds ratio
DM: Diabetes Mellitus
IDDM: Insulin Dependent Diabetes Mellitus
ISA: Insulin Self Administration
NGOs: Non-Governmental Organizations
NIDDM: Non-Insulin Dependent Diabetes Mellitus
SPSS: Statistical Package for Social Sciences
TASH: Tikur Anbasa Specialized Hospital
UKPDS: United Kingdom Prospective Diabetes Study
WHO: World Health Organization
